# Supplementary material for: Mapping the evolution of fertility support policies in China: A content and instrumental analysis
Source: PLoS One. 2025 Oct 9;20(10):e0332137. doi: 10.1371/journal.pone.0332137 (PMC12510515; doi:10.1371/journal.pone.0332137)
Supplement: S1 Appendix — (ZIP) [file pone.0332137.s001.zip › S1 Appendix. 226 original policy documents/67-国务院关于财政社会保障资金分配和使用情况的报告(FBM-CLI.2.5146797).doc]

**国务院关于财政社会保障资金分配和使用情况的报告**

制定机关： [国务院](https://www.pkulaw.com/law/chl?Aggs.IssueDepartment=5&way=textBasic)

公布日期：2022.12.28

施行日期：2022.12.28

时效性： [现行有效](https://www.pkulaw.com/law/chl?Aggs.TimelinessDic=01&way=textBasic)

效力位阶： [国务院规范性文件](https://www.pkulaw.com/law/chl?Aggs.EffectivenessDic=XC0203&way=textBasic)

法规类别： [财政综合规定](https://www.pkulaw.com/law/chl?Aggs.Category=03101&way=textBasic)

国务院关于财政社会保障资金分配和使用情况的报告
--2022年12月28日在第十三届全国人民代表大会常务委员会第三十八次会议上
财政部部长　刘昆

全国人民代表大会常务委员会：
　　受国务院委托，我向全国人大常委会报告财政社会保障资金分配和使用情况。
　　**一、**财政社会保障资金分配和使用基本情况
　　社会保障体系是人民生活的安全网和社会运行的稳定器。党的十八大以来，以习近平同志为核心的党中央对我国社会保障体系建设作出顶层设计，促进我国社会保障事业高质量、可持续发展。各级财政坚持以习近平新时代中国特色社会主义思想为指导，坚决贯彻党中央、国务院决策部署，坚持以人民为中心的发展思想，立足财政职能，按照兜底线、织密网、建机制的要求，积极主动开展工作，加大财政社会保障资金投入，强化资金分配和使用管理，提高资金使用效益，支持建设更加公平更可持续的社会保障体系。
　　（一）调整优化财政支出结构，重点向社会保障领域倾斜。2018-2021年，全国财政社会保障资金累计支出14.88万亿元，年均增幅7.4%，比同期全国财政支出整体增幅高3.8个百分点，占全国财政支出的比重从2018年的14.9%提高到2021年的16.6%。其中，中央对地方社会保障转移支付累计5.89万亿元，年均增幅8.9%，比同期中央对地方转移支付整体增幅高3.3个百分点，占中央对地方转移支付的比重从2018年的18.3%提高到2021年的20%。同时，加强政府性基金预算、国有资本经营预算、社会保险基金预算与一般公共预算的衔接，共同支持做好社会保障工作。在近几年财政收支矛盾突出的形势下，通过持续优化财政支出结构、压减一般性支出，优先保障社会保障等重点支出。突出财政支出的公共性、普惠性，紧紧抓住人民群众最关心最直接最现实的利益问题，推动完善社会保障领域各项财政支出政策。增强财政投入的精准度，坚持保基本、兜底线，强化对社会保障重点领域的支持力度，加快补齐短板弱项，优先保障就业、养老、医疗、社会救助、优抚安置等支出需要，确保党中央、国务院各项决策部署落地见效。
　　（二）支持实施就业优先政策，推动完善就业创业服务体系。2018-2021年，全国各级财政累计安排就业补助资金3658亿元，其中中央对地方转移支付2103亿元，重点支持就业困难群体就业创业，促进各类劳动者公平就业，有效应对中美经贸摩擦、新冠肺炎疫情、经济下行压力等挑战对就业带来的冲击。将就业优先政策置于宏观政策层面并持续强化，把稳就业作为做好“六稳”、“六保”工作和保持经济运行在合理区间的重要指标，推动实现就业与经济发展的良性互动，有力化解就业总量矛盾。支持健全就业创业政策和服务体系，开展职业技能提升行动，面向重点群体和企业职工高质量、大规模开展职业技能培训，有效缓解结构性就业矛盾。推动完善市场化就业政策措施，鼓励引导企业加大力度吸纳高校毕业生就业，支持启动百万就业见习岗位募集计划，积极促进高校毕业生就业创业。支持用好外出务工和就近就业两条渠道，稳定农民工特别是脱贫劳动力就业。健全就业援助机制，加强就业困难群体、零就业家庭成员等就业帮扶。加大对受疫情影响严重的行业、中小微企业、个体工商户的支持力度，实施降低社会保险费率、阶段性减免社会保险费等政策，2018-2021年共减免社会保险缴费2.4万亿元，帮助各类市场主体渡过难关，通过保市场主体来稳就业。经过各方面共同努力，近年来全国城镇就业人数持续增加，由2018年的4.43亿人增加到2021年的4.68亿人，城镇调查失业率水平基本控制在合理水平，就业形势总体稳定，就业结构持续优化，就业质量不断提升。
　　（三）持续提升养老保险覆盖面和待遇水平，确保按时足额发放。截至2021年底，全国基本养老保险参保人数达10.3亿人，其中近3亿人定期领取养老金，人民群众的养老保险权益得到较好保障。持续加大对基本养老保险基金的补助力度，2018-2021年，全国各级财政累计安排养老保险补助支出7.77万亿元，其中中央对地方转移支付3.07万亿元，有力支持各地区养老金按时足额发放。建立退休人员基本养老金合理调整机制，连续提高退休人员基本养老金标准。建立城乡居民基本养老保险待遇确定和基础养老金正常调整机制，推动待遇水平随经济社会发展逐步提高。从2018年起建立并实施企业职工基本养老保险基金中央调剂制度，作为养老保险全国统筹的第一步，调剂比例从3%起步，并以每年0.5个百分点的幅度提高，2021年达到4.5%。2018-2021年中西部地区和老工业基地省份净受益超过6000亿元，有效缓解了地区间养老负担和基金结余分布不均衡等问题。启动实施企业职工基本养老保险全国统筹制度，通过建立养老保险基本要素中央统一管理机制、地方财政补充养老保险基金投入长效机制、工作考核机制等“三个机制”，全国统筹、合理调剂地区间基金余缺，有效压实地方责任，确保养老金按时足额发放。
　　（四）支持构建多层次医疗保障体系，强化基本医保、大病保险、医疗救助三重制度综合保障。2018-2021年，全国各级财政累计安排对基本医疗保险基金的补助和医疗救助资金2.61万亿元，其中中央对地方转移支付1.42万亿元。整合新农合和城镇居民医保，合并实施生育保险和职工医保，提升管理效能。城乡居民医保每年人均财政补助标准从2018年的490元提高到2021年的580元，职工医保和城乡居民医保政策范围内住院费用支付比例分别稳定在80%和70%左右，困难群众经基本医保、大病保险、医疗救助三重保障梯次减负后住院费用实际报销比例达到80%左右，群众就医负担有效减轻。为有效应对疫情冲击，2020年出台新冠肺炎确诊患者免费救治政策，费用由医保基金和财政资金共同负担。支持医保信息化、标准化建设，深化医保支付方式改革、推进药品和医用耗材集中带量采购，加强医保基金监管，控制医疗费用不合理增长。实现所有省份所有统筹地区住院费用和普通门诊费用跨省直接结算全覆盖，2021年惠及1300多万人次，医保基金跨省支付超过600亿元，解决群众跑腿垫资难题。全民医疗保障制度顶层设计不断完善，管理服务水平逐步提升，构建起以基本医疗保险为主体，医疗救助为托底，补充医疗保险、商业健康保险、慈善捐赠、医疗互助共同发展的多层次医疗保障制度体系。
　　（五）完善分层分类社会救助体系，加强困难群众和重点群体救助帮扶。2018-2021年，全国各级财政累计安排困难群众救助、社会福利等支出1.47万亿元，其中中央对地方转移支付5878亿元，支持各地做好困难群众基本生活保障、儿童和老年福利、残疾人康复和托养等工作，全力保障因疫情遇困群众基本生活。健全社会救助和保障标准与物价上涨挂钩的联动机制，将孤儿、事实无人抚养儿童等特殊困难儿童纳入联动机制保障对象范围。推动实施特殊困难老年人居家适老化改造和特困人员供养服务设施（敬老院）改造提升工程。全面实施困难残疾人生活补贴、重度残疾人护理补贴制度。建立残疾儿童康复救助等制度，进一步完善残疾预防和残疾人康复、就业、社会保障等帮扶政策。健全困难群众住房救助制度，对符合规定标准的住房困难的低保家庭、分散供养的特困人员等实施住房救助，帮助解决其住房困难问题。支持做好受灾群众紧急转移安置和过渡性生活救助、因灾遇难人员家属抚慰等工作，加大临时救助力度，切实保障受灾群众基本生活。逐年提升低保等社会救助标准，2021年底全国城市、农村低保平均标准分别达到每人每月711元、530元，比2018年初分别提高170元、172元，增长31.4%、48%。城乡低保平均标准之比从2018年初的1.51：1下降到2021年底的1.34：1，保障水平差距明显缩小。
　　（六）支持完善优抚安置政策，推进建立健全退役军人保障体系。2018-2021年，全国各级财政累计安排抚恤和退役安置资金9487亿元，其中中央对地方转移支付5917亿元，支持各地做好优抚对象等人员抚恤待遇保障和退役军人安置等工作。建立覆盖省、市、县、乡、村五级的退役军人服务保障体系，实现有机构、有编制、有人员、有经费、有保障，打通服务退役军人“最后一公里”。创新退役军人安置方式，建立逐月领取退役金制度，鼓励退役军人就业创业，逐步融入国家社会保障体系。建立完善退役军人和其他优抚对象优待政策制度，制定覆盖各群体的优待目录清单。优抚安置政策不断完善的同时，保障水平明显提高，烈属定期抚恤金年人均标准由2018年的25440元提高到2021年的33860元，年均增长10%左右，优抚对象生活水平显著改善。实行部分退役士兵社会保险费补缴政策，中央财政对基本养老保险单位缴费部分给予适当补助。在建党百年之际，中央财政专门安排资金支持实施全国县级以下英雄烈士纪念设施整修工程，将全国56万座烈士纪念设施纳入修缮保护范围。
　　总的看，随着财政社会保障资金支出的不断增加和社会保障体制机制改革的不断深化，近年来我国社会保障体系建设进入了快车道。社会保障体系更加健全，以社会保险为主体、包括社会救助、社会福利、社会优抚等制度在内，功能完备的社会保障体系基本建成，为更好实现幼有所育、学有所教、劳有所得、病有所医、老有所养、住有所居、弱有所扶等民生保障目标奠定了坚实的制度基础；养老保障、医疗保障、城乡低保、抚恤优待等重点社会保障项目基本实现全覆盖或“应保尽保”，形成了世界上规模最大的社会保障网；社会保障各项目的保障水平明显提升，与我国经济社会发展阶段总体比较协调，社会保障对象特别是困难群众的基本生活得到较好保障。社会保障制度建设的丰硕成果，使人民群众的获得感、幸福感、安全感更加充实、更有保障、更可持续。
　　这些成绩的取得，是以习近平同志为核心的党中央坚强领导的结果，是习近平新时代中国特色社会主义思想科学指引的结果，是各级人大及其常委会监督指导的结果，是各地区各部门共同努力的结果。经过长期努力特别是新时代十年的不懈奋斗，我国深化了对社会保障体系建设规律的认识，成功建设了具有鲜明中国特色的社会保障体系，必须坚持和发展成功经验，不断总结，不断前进。一是坚持和加强党的全面领导，不折不扣贯彻习近平总书记重要讲话和重要指示批示精神，坚决落实党中央决策部署，发挥党的领导和社会主义制度的政治优势。二是坚持人民至上、共同富裕，把增进民生福祉、促进社会公平作为发展社会保障事业的根本出发点和落脚点，使改革发展成果更多更公平惠及全体人民。三是坚持制度引领，围绕全覆盖、保基本、多层次、可持续等目标加强社会保障体系建设。四是坚持与时俱进，用改革的办法和创新的思维解决发展中的问题，坚决破除体制机制障碍，推进社会保障体系建设，推动社会保障事业不断前进。五是坚持实事求是，既尽力而为、又量力而行，把提高社会保障水平建立在经济和财力可持续增长的基础上，不脱离实际、超越阶段，确保社会保障制度安全可持续。

　　**二、**加强财政社会保障资金管理的主要措施和存在的问题
　　按照党中央、国务院决策部署，财政部会同有关部门认真落实[预算法](https://www.pkulaw.com/chl/4fef1f2f1d7b7441bdfb.html?way=textSlc)及其实施条例等法律法规规定，积极采取措施加强财政社会保障资金管理，建立健全管理机制，着力增强资金分配的科学性、规范性和有效性，不断提高财政资金的使用效益。
　　（一）建立健全财政社会保障支出责任分担机制。根据基本公共服务等领域中央与地方共同财政事权和支出责任划分改革方案要求，增强地方基本公共服务保障能力。充分考虑我国各地经济社会发展不平衡、基本公共服务成本和财力差异较大等国情，中央财政承担的基本公共服务支出责任体现地区差别，重点向中西部等困难地区倾斜。对于城乡居民基本医疗保险，中央财政按照五档进行补助，其中对西部地区按最高档80%补助，对中部地区按第二档60%补助。对于城乡居民基本养老保险，中央财政对中西部地区按国家确定的基础养老金标准给予全额补助，对东部地区补助50%。对于基本公共就业服务、医疗救助、困难群众救助、残疾人服务等事项，主要依据地方财力状况、保障对象数量和工作绩效等因素确定中央支出责任。企业职工基本养老保险全国统筹制度明确中央与地方支出责任，适当加强中央事权，保持中央财政投入力度不减；压实地方政府支出责任，建立健全地方财政补充养老保险基金投入长效机制，防范基金运行风险向中央财政转移集聚。将义务兵家庭优待由地方财政事权调整为中央和地方共同财政事权，有效解决军地反映强烈的义务兵家庭优待金标准差异较大、地方财政负担不平衡等问题。
　　（二）完善财政社会保障资金管理制度办法。认真贯彻执行[预算法](https://www.pkulaw.com/chl/4fef1f2f1d7b7441bdfb.html?way=textSlc)及其实施条例等法律法规规定，推动完善社会保障管理制度体系，大力推进社会保障法制化。修订完善《[社会保险基金财务制度](https://www.pkulaw.com/chl/e4e06e49beb752e2bdfb.html?way=textSlc)》，形成覆盖全部险种、完整统一的社会保险基金财务管理制度。制定完善基本养老保险、城乡居民医保、困难群众救助、农村危房改造等十多项补助资金管理办法，规范资金分配、下达、使用等全链条各环节，同时明确使用管理要求，加大重复参保审核力度，严厉打击欺诈骗取财政资金行为。加强基础数据和项目库管理，提高资金分配的规范性精准性。
　　（三）建立财政资金直达机制。认真落实党中央、国务院部署，将社会保障领域符合条件的中央对地方转移支付资金全部纳入直达机制管理。建立健全直达资金监控体系，加强部门协同联动，强化从资金源头到使用末端的全过程、全链条、全方位监管，资金监管“一竿子插到底”，确保资金直达使用单位、直接惠企利民，防止挤占挪用、沉淀闲置，提高财政资金使用的有效性和精准性。通过加强社会保障领域财政资金直达管理，资金支出进度和使用效率显著提升，有效支持了地方做好社会保障和民生兜底等工作。
　　（四）加强预算绩效管理。相关部门制定出台《社会保险基金预算绩效管理办法》，完善绩效评价指标，形成预算编制有目标、预算执行有监控、预算完成有评价、评价结果有反馈、反馈结果有应用的预算绩效管理闭环，加快构建全方位、全过程、覆盖各险种的社会保险基金预算绩效管理体系。强化部门间协调配合，主动扩大重点绩效评价项目范围，开展全过程绩效评价，加强评估结果应用；对困难群众救助、农村危房改造、医疗救助、县级以下烈士纪念设施等补助资金开展绩效评价，建立绩效评价结果与预算安排、政策调整挂钩机制，强化绩效硬约束，削减低效无效资金，真正做到“花钱要问效、无效要问责”。
　　总的看，当前财政社会保障资金投入使用呈现出总量持续增长、机制逐步健全、效益日益提升、成效不断显现的良好态势。同时，也要清醒地认识到，随着我国社会主要矛盾发生变化和城镇化、人口老龄化、就业方式多样化加快发展，叠加百年变局和世纪疫情给我国经济社会发展和民生保障带来的冲击，我国社会保障体系仍存在一些突出问题和短板弱项，社会保障支出压力和社会保障资金管理难度进一步加大。一是社会保障部分项目的职能定位不够明确，政府与市场、单位与个人的责任边界不够清晰，部门间还存在职责分散或交叉现象。多层次社会保障体系发展还不均衡，政府主导并负责管理的基本保障“一枝独大”，而市场主体和社会力量承担的补充保障发育不够，养老保险第二支柱中的企业年金覆盖率较低，第三支柱发展不够，慈善事业发展不充分。社会保障部分领域政府间支出责任划分不够清晰，中央与地方财政事权和支出责任划分改革相关部署还需进一步推进落实。二是社会保障体系与系统集成、协同高效的目标还有差距，在项目与项目、城市与农村、东中西不同区域和不同群体之间统筹协调还不够，部分农民工、灵活就业人员、新业态从业人员等人群没有纳入社会保障体系，存在“漏保”、“脱保”、“断保”等情况。部分群体社会保障水平偏低。三是社会保障制度的安全可持续压力加大，在人口老龄化背景下，职工基本养老保险在职职工与退休人员的抚养比不断下降，随着参保人平均领取待遇年限的延长，社会保障制度的安全可持续发展面临严峻挑战。社会保险基金、企业年金、职业年金等保值增值和安全监管难度持续加大，一些地方社保基金存在“穿底”风险。四是社会保障资金管理能力和水平亟待提升，部门之间、中央与地方之间还存在“信息孤岛”和“数据鸿沟”，尚未实现基础信息及时共享，容易引发“跑冒滴漏”问题。预算约束软化，部分地方还存在违规调整社会保险基金预算、财政对社会保险基金的补助未及时拨付到账等情况。财政社会保障支出标准化建设有待进一步规范，科学、公平、可持续的待遇项目清单和支出标准体系尚未成型。同时，部分资金使用单位绩效管理不到位，存在相关制度不完善、预算执行率不高、资金连年结转、绩效评价结果应用不够等问题。
　　针对这些问题和不足，财政部将会同相关部门切实采取措施，逐步加以解决。

　　**三、**下一步工作考虑
　　党的二十大报告提出，必须坚持在发展中保障和改善民生，鼓励共同奋斗创造美好生活，不断实现人民对美好生活的向往。财政部将会同民政部、人力资源社会保障部、退役军人部、国家医保局等部门全面贯彻落实党的二十大精神，以习近平新时代中国特色社会主义思想为指导，不断提高政治判断力、政治领悟力、政治执行力，坚持目标导向、问题导向，坚持系统观念、战略眼光，增强风险意识，围绕健全覆盖全民、统筹城乡、公平统一、安全规范、可持续的多层次社会保障体系，科学把握经济发展和社会保障水涨船高的关系，坚持全国一盘棋，坚持制度的统一性和规范性，认真谋划做好下一步的财政社会保障工作，推动社会保障体系更加成熟、定型。
　　（一）加强财政资源统筹，加大财政保障力度。大力优化财政支出结构，继续增加社会保障领域投入，加大对中西部地区和农村社会保障事业发展的支持力度，既持续提高社会保障水平，又逐步缩小城乡社会保障待遇差距。落实好稳岗促就业等政策措施，继续支持大规模实施职业技能培训，推动提高培训质效，支持技工教育发展，保持就业大局稳定。健全养老金合理调整机制，综合考虑工资增长、物价指数和财力状况等因素，合理提高退休人员基本养老金水平，逐步提高城乡居民基础养老金标准，让广大退休职工、城乡老年居民更好共享经济社会发展成果。同时，引导激励城乡居民早参保、多缴费，增加城乡居民基本养老保险个人账户资金积累。支持规范发展多层次养老保险体系，大力发展企业年金、职业年金，规范发展第三支柱养老保险，落实好推动个人养老金发展的政策措施。逐步提高城乡居民医保财政补助标准，夯实医疗救助托底保障，支持医疗保障服务能力建设。完善基本医保筹资分担和调整机制，研究应对人口老龄化的多渠道医保筹资政策。完善和规范职工大额医疗费用补助、企业补充医疗保险等制度，鼓励商业健康保险、慈善捐赠、医疗互助等协调发展。增强社会救助兜底功能，指导地方结合财力状况合理制定低保标准和特困人员供养标准并建立动态调整机制。支持加快构建多层次养老服务体系，合理划分个人、家庭、社会、政府等在养老服务中的责任边界，加大制度创新力度，增加居家和社区养老服务供给，健全居家社区机构相协调、医养康养相结合的养老服务体系。适当提高优抚对象等人员抚恤和生活补助标准，并向参加革命早、服役贡献大、伤残等级高的重点对象适当倾斜。合理确定退役士兵职业教育和技能培训补助标准。加强社会保险基金、职业年金和企业年金的投资运营管理，支持做大做强全国社会保障基金，提升基金的保值增值能力。
　　（二）积极完善制度政策体系，支持体制机制改革创新。坚持人民至上，把增进民生福祉、促进社会公平作为发展社会保障事业的出发点和落脚点，在法治化轨道上推进社会保障精细化管理，提升社会保障治理效能。支持强化就业优先政策，健全就业促进机制。完善重点群体就业支持体系，加强困难群体就业兜底帮扶。支持统筹城乡就业政策体系，破除妨碍劳动力、人才流动的体制和政策弊端，消除影响平等就业的不合理限制和就业歧视。健全灵活就业人员社会保障政策，强化激励约束机制，保障好灵活就业人员社会保障权益。支持完善企业职工基本养老保险全国统筹制度，开展省级政府养老保险工作考核，增强基本养老保险制度的规范性、可持续性。支持发展多层次、多支柱养老保险体系。完善基本医疗保险门诊共济保障机制，优化职工医保统筹基金和个人账户结构。支持全面做实基本医保市地级统筹，鼓励有条件的省份推进省级统筹，强化基层政府在医保支付管理、基金监管等方面的主体责任。扩大社会保险覆盖面，健全基本养老、基本医疗保险筹资和待遇调整机制，推动基本医疗保险、失业保险、工伤保险省级统筹。推进药品和高值医用耗材集中带量采购，深化医保支付方式改革，完善大病保险和医疗救助制度，落实异地就医结算。支持健全分层分类的社会救助体系，完善最低生活保障和特困人员救助供养制度，加强社会救助政策和巩固拓展脱贫攻坚成果制度性安排的有效衔接，进一步提高社会救助兜底保障能力。加强长期护理保险制度试点总结评估，支持逐步形成适合我国国情的长期护理保险制度框架。支持健全养老服务体系，重点保障老年人面临家庭和个人难以应对的失能、残疾、无人照顾等困难时的基本需求。完善退役士兵职业教育和技能培训支持政策，增强退役士兵就业创业能力。发挥好社会保障在应对疫情影响方面的积极作用，完善我国社会保障针对突发重大风险的应急响应机制。
　　（三）坚持尽力而为、量力而行，确保政策精准可持续。统筹需要和可能，尽力而为，量力而行，推动社会保障政策与经济发展阶段相适应。在预算安排上优先保障和重点支持国家出台的统一社会保障政策，确保不折不扣抓好落实。在此基础上，推动地方对自行出台的社会保障政策开展财政承受能力评估，加强事前论证和风险评估，全面分析对财政支出的当期和长远影响，对于评估认定不具备实施条件或存在较大风险隐患的民生政策，一律不得实施，进一步增强社会保障政策措施的精准性、有效性和可持续性，防范过高承诺、过度保障。推动建立社会保险精算制度，提高风险识别应对能力，增强重大改革举措和政策制定的科学性、有效性，提升制度的可持续性。加强社会保障项目之间、部门之间、中央与地方之间的统筹协调，合理划分政府与市场、单位与个人的责任，切实增强合力。完善社会保障支出清单管理制度，明确相关政策名称、保障范围、支出标准、备案流程等，并逐步与预算编制挂钩。
　　（四）健全资金分配管理机制，提高财政资金使用效益。按照党中央、国务院决策部署，直面人民群众急难愁盼问题，优化支持方式，突出工作重点，聚焦群众最关切的养老、医疗、就业等领域集中发力，提高财政资金分配的科学性精准性。完善中央财政养老保险补助资金分配机制，对省级政府养老保险工作进行考核，根据考核结果适当调整各省份补助金额，体现奖惩，引导地方进一步规范基金收支管理。完善社会保障领域中央对地方转移支付资金直达机制，加快资金下达速度，强化对直达资金的库款保障，健全全流程监控体系，确保安全规范使用直达资金。认真落实全国人大审查意见，提升财政社会保障资金效能，更加注重精准、可持续。加强社会保障资金审计等监督结果应用，推动做好审计查出问题、执行监控发现问题等的整改工作。推进预算绩效管理，强化中央转移支付与绩效评价结果挂钩机制，对于问题突出的地方，相应扣减补助或者不予安排，加大对资金使用绩效较好地区的支持力度，更好发挥预算绩效管理在优化财政资源配置方面的作用。
　　委员长、各位副委员长、秘书长、各位委员，长期以来，全国人大常委会高度重视财政工作，对财政社会保障工作给予大力支持和悉心指导，有力推动了社会保障事业发展。我们要更加紧密地团结在以习近平同志为核心的党中央周围，以习近平新时代中国特色社会主义思想为指导，深刻领悟“两个确立”的决定性意义，增强“四个意识”、坚定“四个自信”、做到“两个维护”，认真贯彻党中央、国务院决策部署，按照全国人大常委会审议意见，不断完善财政支持政策，着力保障和改善民生，为全面建设社会主义现代化国家、全面推进中华民族伟大复兴而团结奋斗！

#### 本篇引用的法规

##### 中央法规

[中华人民共和国预算法(2018修正)](https://www.pkulaw.com/chl/4fef1f2f1d7b7441bdfb.html?way=textRightFblx)

[社会保险基金财务制度](https://www.pkulaw.com/chl/e4e06e49beb752e2bdfb.html?way=textRightFblx)

©北大法宝：（[www.pkulaw.com](https://www.pkulaw.com/)）专业提供法律信息、法学知识和法律软件领域各类解决方案。北大法宝为您提供丰富的参考资料，正式引用法规条文时请与标准文本核对。 欢迎查看所有[产品和服务](http://www.pkulaw.net/)。
[法宝快讯： 如何快速找到您需要的检索结果？ 法宝 V6 有何新特色？](http://www.pkulaw.com/helps/69.html)

扫描二维码阅读原文

原文链接：<https://www.pkulaw.com/chl/90faef1ea993472bbdfb.html>
